# CMV Enteritis Causing Massive Intestinal Hemorrhage in an Elderly Patient

**DOI:** 10.1155/2010/385795

**Published:** 2010-07-12

**Authors:** Mihaiela Morunglav, Ivan Theate, Gilles Bertin, Philippe Hantson

**Affiliations:** ^1^Department of Intensive Care, Cliniques St-Luc, Université Catholique de Louvain, 1200 Brussels, Belgium; ^2^Department of Pathology, Cliniques St-Luc, Université Catholique de Louvain, 1200 Brussels, Belgium; ^3^Department of Radiology, Clinique Ste-Anne, 1070 Brussels, Belgium

## Abstract

*Background*. Cytomegalovirus (CMV) disease is rare in previously immunocompetent patients. We report a case of CMV enteritis complicated by massive intestinal bleeding. *Case History*. A 72-year-old immunocompetent patient was admitted for diarrhea and abdominal pain. Aspecific pattern of duodenitis was found at abdomen computed tomography and on biopsies during endoscopy. A diagnosis of vasculitis was suspected on the basis of the clinical and biological course (skin lesions, arthralgias, proteinuria, low complement C3 and C4 fractions, etc.) and pulse steroid therapy was prescribed. The patient developed multiple episodes of intestinal bleeding with shock and required urgent laparotomy. Jejunitis due to CMV vasculitis was proven by histological examination of the operative specimen. Treatment with ganciclovir was initiated. No bleeding recurrence was noted. No other lesions from CMV infection were observed. The patient died from unrelated complications. *Discussion*. CMV enteritis is a rare cause of intestinal bleeding particularly in previously immunocompetent patients. Aging could be accompanied by a relative immune weakness and specific antiviral therapy seems to be indicated.

## 1. Introduction

Cytomegalovirus (CMV) enteritis is mostly associated with patients positive for human immunodeficiency virus or immunosuppressed transplant patients. Involvement of small bowel is rare. We report a case of CMV jejunitis, without colon involvement, complicated by massive intestinal bleeding in a previously immunocompetent elderly patient.

## 2. Case Report

A 72-year-old man was admitted in a first hospital for diffuse abdominal pain and diarrhea. This patient had no history of malignancy, HIV, diabetes or other chronic disease, and was never treated by immunosuppressive drugs. He was chronically treated for Parkinson disease, asbestosis, paroxystic atrial fibrillation, hyperuricemia and hypercholesterolemia. His current medications were allopurinol, amiodarone, levodopa and simvastatin. He was not under antiplatelet or anticoagulant therapy prior to admission. On physical examination, the patient was apyretic, with supple abdomen and no rebound tenderness. Peripheral oedema was noted. Routine laboratory investigations revealed: C-reactive protein (CRP) 16.4 mg/dL, white blood cell count 18,900/mm^3^, platelet count 530,000/mm^3^, INR 1.15, fibrinogen 396 mg/dL, proteins 3.8 g/dL, albumin 1.9 g/dL, urea 70 mg/dL, creatinine 0.80 mg/dL. A moderate proteinuria was noted (1.267 g/24 hr) without urinary casts.

**Figure 1:**
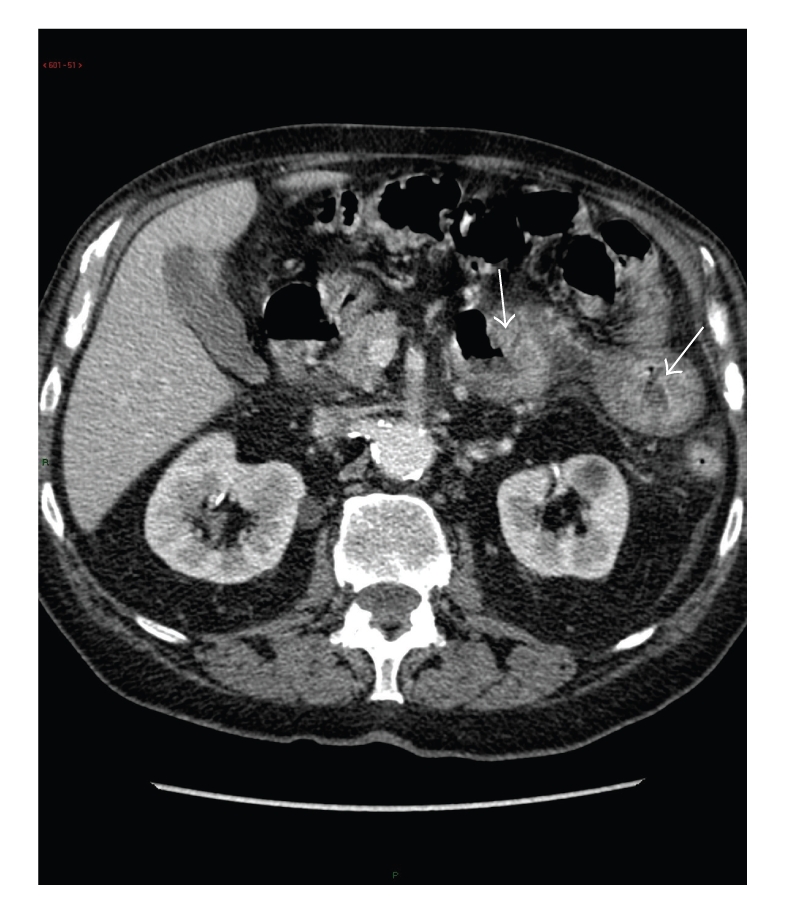
Abdomen computed tomography after intravenous contrast. Significant thickening of the Treitz angle and proximal jejunum (arrows), with infiltration of the loco-regional fat.

The patient was investigated by abdomen computed tomodensitometry (CT) and upper and lower endoscopy. The abdomen CT with intravenous contrast disclosed a significant thickening of the wall of the third, fourth duodenum, and of the proximal part of the jejunum with infiltration of the loco-regional fat (Figure 1).

Esogastroscopy revealed a proliferative aspect in the duodenum. Duodenal biopsy showed an infiltration of the mucosa by polynucleated cells, but no ulceration was evident. The lower endoscopy was negative.

A few days later, ecchymotic lesions appeared on the limbs and the patient started also to complain from diffuse arthralgias and weakness in the upper and lower limbs. Electromyography was consistent with an acute sensorimotor polyneuropathy.

Serological investigations revealed: rheumatoid factor (-), anti-nuclear antibodies (-), anti-neutrophilic cytoplasmic antibodies (-), cryoglobulin (-), low C3 and C4 fractions of complement. Serological tests were negative for hepatitis B and C, EBV and HIV.

Due to the possibility of a systemic inflammatory disease combining skin lesions, arthralgia, polyneuropathy, proteinuria, high CRP with low complement fractions, a pulse steroid therapy was proposed on day 14 for a presumptive diagnosis of vasculitis, either microscopic polyangiitis (MPA) or polyarteritis nodosa (PAN). However, no skin or sural nerve biopsies were performed. The steroids loading dose was 1 g methylprednisolone, followed by 2 mg/kg/day for one week, and then 4 mg/day.

**Figure 2:**
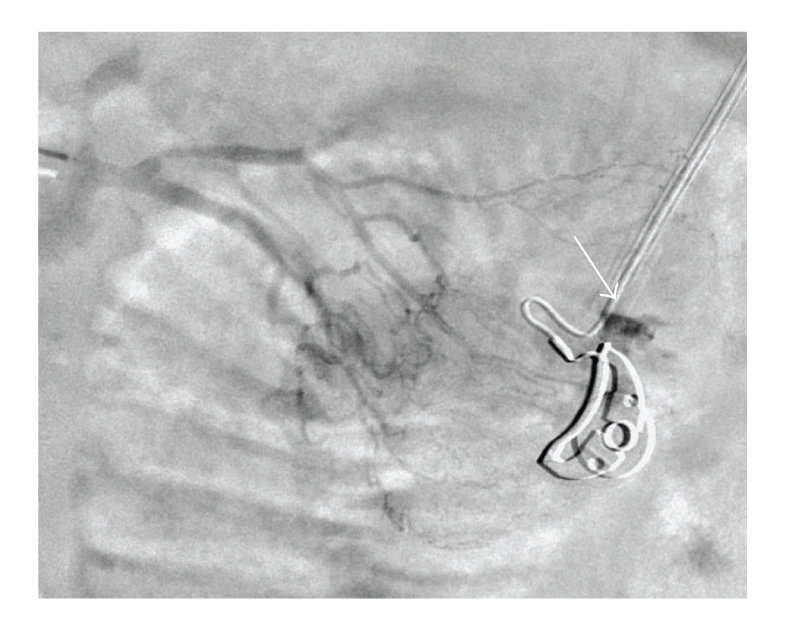
Angiography demonstrating active bleeding (arrow) in one of the branchs of the jejunal arteries.

The patient was referred to the intensive care unit (ICU) of our university hospital on day 15 for gastrointestinal bleeding with melena. Despite blood and platelets transfusions, he remained with a low arterial blood pressure at 90/65 mmHg and hemoglobin concentration remained below 0.4 g/dL. Urgent gastroscopy failed to reveal any lesion. Angiography demonstrated that the bleeding originated from a jejunal branch and microcatheter embolization was successful initially (Figure 2). However, two major episodes of lower intestinal bleeding occurred again within 48 hours after the procedure. On day 19, conventional enteroscopy revealed proximal jejunal ulcer with visible vessel and endoscopic clipping was performed. During the ICU stay, the patient was fed by intravenous parenteral nutrition and received daily intravenous vitamin K1 supplements. Despite optimization of the coagulation, rebleeding was noted on day 26 (hemoglobin 0.37 g/dL), but abdomen CT and angiography with direct intra-arterial provocative papaverine administration were not able to identify the origin. Scintigraphy with 99mTc labeled erythrocytes suggested that the origin was proximal small bowel. After a recurrence of hemorrhagic shock on day 28, the patient underwent urgent laparotomy. Active jejunal bleeding was found leading to segmental resection. Lower intestinal bleeding was definitely stopped after surgery.

**Figure 3:**
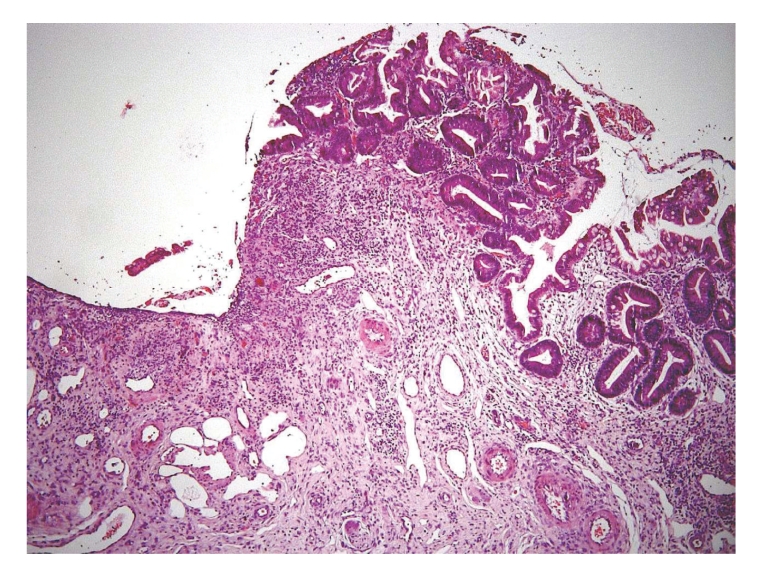
Microscopic examination (hematoxylin-eosin, x150) of the operative specimen. Ulcerative ileitis, the mucosa is replaced by a granulation tissue.

**Figure 4:**
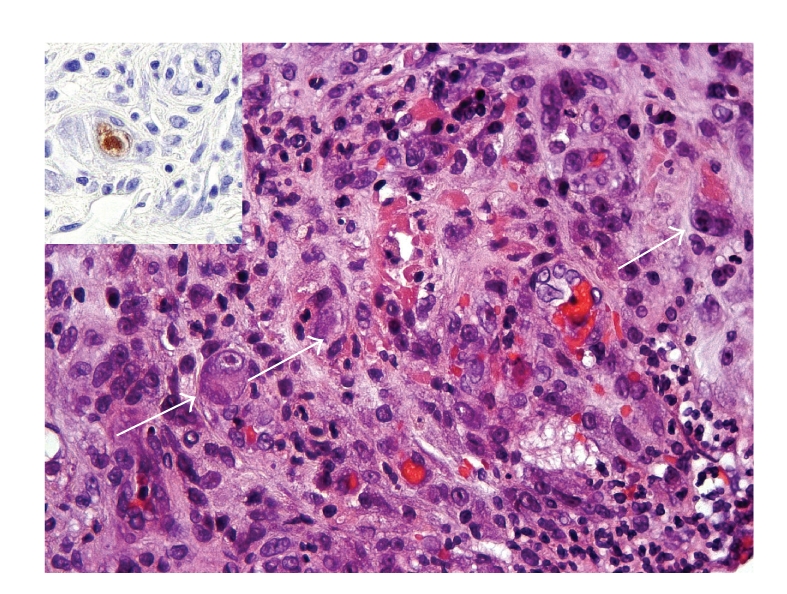
High-power microscopic examination (hematoxylin-eosin, x400) revealed vasculitis. Some endothelial cells present cytomegaly, suggestive of CMV infection (arrows). The presence of CMV in the jejunum was demonstrated by immunohistochemistry (inset; immunoperoxidase, x400).

Histology of the jejunal resection showed a severe mucosal necrosis with loss of villi (Figure 3). High-power examination revealed thrombosis and fibrinoid necrosis suggestive of vasculitis. Some of the endothelial cells presented the characteristic cytopathogenic aspect of CMV infection (Figure 4).

CMV antigenemia determined by real-time PCR in blood was weakly positive (5200 DNA copies/mL).

Multiple skin biopsies (hands, arms, legs) had also been performed on day 20. The histological picture was consistent with a diagnosis of leukocytoclastic vasculitis, but was not specific. No CMV inclusion was noted. Direct immunofluorescence showed deposits of IgG, IgA, IgM and C3 fraction of complement. 

There was no evidence of CMV-related retinitis, pneumonia, hepatitis or bone marrow depression; the gastrointestinal involvement was restricted to the small bowel.

Ganciclovir (200 mg every 12 h) was given intravenously for 14 days to treat CMV disease. CMV antigenemia was negative at the end of the treatment. The patient had been maintained on low doses of oral methylprednisolone (4 mg/day); steroids were discontinued after 2 months. Other complications, mainly bacterial pneumonia and sepsis, occurred during hospital stay. After a significant recovery, the patient was discharged from the ICU on day 58, but died on day 159 from unrelated cardiorespiratory complications. Postmortem examination was not obtained.

## 3. Discussion

CMV enteritis is a rare disease occurring mainly in immunocompromised patients. Symptoms of CMV enteritis may range from mild anorexia to obvious hemorrhage and perforation. The pathogenesis of CMV enteritis is related to the infection of vascular endothelial cells [1, 2]. When CMV infects small bowel, it tends to invade a particular region rather than becoming a panenteric process [3, 4]. Signs of CMV vasculitis may be also observed in other organs (central nervous system, lungs, skin, etc.) [5].

A severe CMV disease is possible in patients receiving immunosuppressive agents for a chronic inflammatory disease, even at relatively low doses [6, 7]. However, in most of the cases, other medications were given in addition to steroids and the duration of therapy is long. Our patient was admitted with clinical manifestations consistent with gastrointestinal CMV infection, but as in previous cases of small bowel involvement, the diagnosis was made with some delay [1, 8]. The patient was not initially tested for CMV serology. Serologies, however, are not sufficient to make the diagnosis of CMV-associated disease. Viremia can be detected by PCR, but compartimentalized disease can occur with minimally detectable or undetectable virus in the blood. CMV infection could not be demonstrated initially on duodenal biopsies, but well on the jejunal operative specimen. Biopsies should be done deep enough to obtain endothelial cells and fibroblasts within the lamina propria because the virus may not be present in the more superficial epithelial tissue. As the clinical, radiological and endoscopic signs appeared before any steroid therapy, we suspect that a community-acquired CMV infection or reactivation due to age-related impaired immunity preceded the short course of steroid therapy. 

The mortality rate of CMV vasculitis is high for patients in a condition of immunosuppression, while the prognosis is better for those who are immunocompetent [8]. Galiatsatos et al. identified risk factors that might influence the prognosis of CMV colitis in immunocompetent patients [9]. They found only 43 cases from a literature search performed from 1980 to 2003. There was a higher mortality among male patients older than 55 years and in patients with co-existing immune-modulating conditions, chiefly renal failure and diabetes mellitus. Spontaneous resolution is possible in younger patients, even in the absence of specific antiviral therapy. No data are specifically available for CMV enteritis, but the risk factors are probably similar. Our patient had no significant co-morbidity, but we cannot exclude that even a short course of steroids could have amplified CMV disease. In a 35-year-old man who developed fulminant hepatitis from unknown origin, and who had received pulse steroid therapy followed by decreasing doses of oral methylprednisolone, small-bowel hemorrhage due to CMV vasculitis occurred approximately 8 weeks after disease onset and required ileal resection [10]. 

The involvement of CMV infection in the other manifestations (cutaneous necrotic lesions, arthralgias, neuropathy, proteinuria, etc.) remains speculative [11]. It cannot be excluded that a systemic vasculitis was triggered by CMV infection, making important the differential diagnosis between virus-associated vasculitis and idiopathic MPA or PAN [12]. This would result in a less aggressive cytotoxic treatment for the former manifestations.

In the present observation, testing for anti-nuclear, anti-neutrophilic cytoplasmic antibodies and cryoglobulin was negative and the systemic manifestations resolved after ganciclovir therapy and maintenance of low doses prednisolone. The presence of skin lesions could be a marker of a poorer prognosis [5]. Cutaneous vasculitis may result from viral infection of endothelial cells, but as in our case, vasculitis without local evidence of virus can also be noted; the presence of immune complexes deposits suggests an immune-mediated process. 

Massive bleeding from CMV-related small-bowel ulcers is an extremely rare occurrence [13–16]. As in our case, symptoms preceding hemorrhage or perforation are usually vague. The first report of CMV enteritis causing segmental ischemia and massive intestinal hemorrhage in an immunocompromised patient appeared in 2001 [2]. Additionally, small-bowel hemorrhage after CMV ileitis was diagnosed in a 73-year-old man receiving oral prednisolone for approximately 2 months to treat nonsystemic vasculitic neuropathy [17]. Fatality from hemorrhage was also noted in a 66-year-old man who had only type 2 diabetes as an immune-modulating condition [14]. Due to the location of the bleeding, the diagnosis is always difficult and could only be made either by enteroscopy or angiography.

Ganciclovir remains the treatment of choice of gastrointestinal CMV disease in immmunocompromised patients and no studies have reported the efficacy of foscarnet in patients without AIDS. However, the overall response to ganciclovir in immunocompetent patients is unknown. Withdrawal of immunosuppressive drugs is also essential when possible.

In conclusion, this case illustrates the difficult early diagnosis of segmental CMV enteritis in an immunocompetent patient and also the risks of high doses steroids therapy when the apparent diagnosis of vasculitis is only presumptive. Endoscopic studies with biopsies are essential together with the determination of CMV antigenemia. The tests must be repeated if the initial results are inconclusive. Surgical treatment should remain exceptional in case of massive gastrointestinal bleeding that could not be controlled by selective embolization. Due to the high risk of complications and mortality of CMV enteritis in the elderly, all the patients must be offered antiviral treatment as soon as possible. The duration of the treatment should be guided by the clinical course and by the negativity of CMV antigenemia.
